# Dorzagliatin shows potential in preventing cognitive impairment in diabetes: evidence from Mendelian randomization analysis and animal study

**DOI:** 10.3389/fendo.2025.1755359

**Published:** 2026-01-23

**Authors:** Jiangxia Ni, Ke Wang, Lingge Feng, Chuyi Wang, Changhong Li, Yelin Chen, Li Chen

**Affiliations:** 1 Hua Medicine (Shanghai) Limited, Shanghai, China; 2 Interdisciplinary Research Center on Biology and Chemistry, Shanghai Institute of Organic Chemistry, Chinese Academy of Sciences, Shanghai, China

**Keywords:** dorzagliatin, glucokinase activator, glucose homeostasis, Goto Kakizaki rat, memory loss, Mendelian randomization, neurodegenerative disorders

## Abstract

**Aim:**

Dorzagliatin is a glucokinase (GK) activator, restoring glucose homeostasis in type 2 diabetes. We investigated the effects of dorzagliatin on cognitive traits using Mendelian randomization (MR), with validation in a spontaneous diabetic rat model.

**Methods:**

A two-sample MR study was conducted to investigate the causal effects of GK activation on neurodegenerative traits. Utilizing genome-wide association study summary statistics, we selected independent genetic variants of *GCK* (encodes GK) associated with lower HbA1c as instrumental variables to mimic GK activation. An animal validation study was further performed. Goto Kakizaki rats and Wistar rats were treated with vehicle or low-dose dorzagliatin (8mg/kg, i.g, bid, below the therapeutic level) for 36 weeks. Morris water maze (MWM) test, western blot analyses were carried out to investigate the neuroprotective effects of dorzagliatin and explore the potential mechanisms.

**Results:**

Genetically mimicked GK activation causally decreased risk of memory loss (OR 0.21 per 1% lower HbA1c, 95% CI 0.05-0.91) and was associated with higher scores of prospective memory task, symbol digit substitution task and intelligence. MR results also implied that GK activation had cognitive protective effects not solely attributed to glucose-lowering. Low-dose dorzagliatin treatment in young Goto Kakizaki rats prevented spatial memory impairment occurred in adulthood in the MWM test. It also significantly prevented the reduced expression of insulin receptors, glucose transporters, and synaptic proteins in the brains of Goto Kakizaki rats.

**Conclusions:**

Dorzagliatin protects against cognitive impairment under diabetes conditions. Maintaining glucose homeostasis directly regulates insulin pathway and glucose uptake, as well as enhances neurotransmission processes in the hippocampus. These findings not only highlight dorzagliatin as a promising therapeutic option for preventing diabetes-associated cognitive decline but also provide critical mechanistic insights into the role of GK modulated glucose homeostasis in preserving brain function, offering a potential translational strategy for clinical intervention.

## Introduction

Type 2 diabetes (T2D) has become a rapidly growing health concern worldwide, leading to a rise in disability rates and premature death. Accumulating evidence positions T2D as an independent risk factor for accelerated cognitive decline and neurodegenerative disorders (1). It is reported that mild cognitive impairment (MCI) is common in T2D patients with 45% incidence rate (2). Diabetes associated cognitive dysfunction (DACD) is characterized by progressive memory loss, executive function impairment, and deficiency in information process speed (3, 4). To date, therapeutic targets for this neurocognitive complication remain elusive, and no disease-modifying treatments have been approved.

Recent studies suggest that multiple mechanisms contribute to DACD, including metabolic dysfunctions (e.g., aberrant abnormal amyloid, hyperglycemia, hypoglycemia, and insulin resistance) and vascular impairments (e.g., endothelial impairments, atherosclerosis, hypertension, stroke, and inflammation) (5, 6). These risk factors are closely correlated with the loss of systemic glucose homeostasis. The brain, although accounting for only 2–3% of total body mass, consumes approximately 20% of the body’s oxygen and 25% of its glucose supply, underlining its exceptional energy demands (7). Despite being one of the most energy-demanding organs in the body, the brain mostly lacks intrinsic energy reserves. Glucose homeostasis is essential for supporting neuronal communication and cognitive processes including learning and memory (8). Glucose-derived ATP is critical for maintaining ion gradients required for electrical signaling and serves as a precursor for neurotransmitters (9). Notably, disruptions in cerebral glucose supply and utilization are among the earliest detectable abnormalities in cognitive impairment related to diabetes and Alzheimer’s disease.

Glucokinase (GK) acts as a glucose sensor in pancreas and intestine endocrine cells regulating glucose-stimulated hormone secretion, which plays a central role in systemic glucose homeostasis. Impaired GK expression and function have been documented in patients with diabetes, leading to reduced glucose sensitivity and defective insulin and GLP-1 secretion, as well as impaired glycogen synthesis. Beyond its peripheral actions, GK is also involved in glucose sensing within neurons of the ventromedial hypothalamus, a pivotal center for feeding behavior and glucose regulation (10, 11). Dorzagliatin, a first-in-class GK activator (GKA) approved in China in 2022 for T2D, restores glucose homeostasis by repairing the glucose sensor function of GK in multiple glucose-metabolizing tissues (12, 13). We hypothesize that by ameliorating impaired glucose homeostasis, dorzagliatin may potentially offer preventative or therapeutic benefits against metabolic impairment induced cognitive disorders. The current study investigates the potential genetic evidence supporting the GK activation on neurodegenerative effects by Mendelian randomization (MR) frameworks. MR leverages genetic variants randomly assigned at conception as proxies to infer causal relationships between exposures and outcomes, thereby reducing confounding (14). We further validate these genetic insights using the Goto Kakizaki rat model, a non-obese model that develops spontaneous diabetes with age, exhibiting features such as mild hyperglycemia, insulin resistance, progressive beta-cell failure, and cognitive deficits accompanied by impaired hippocampal synaptic development (15, 16).

## Methods

### Study design

**Figure 1:**
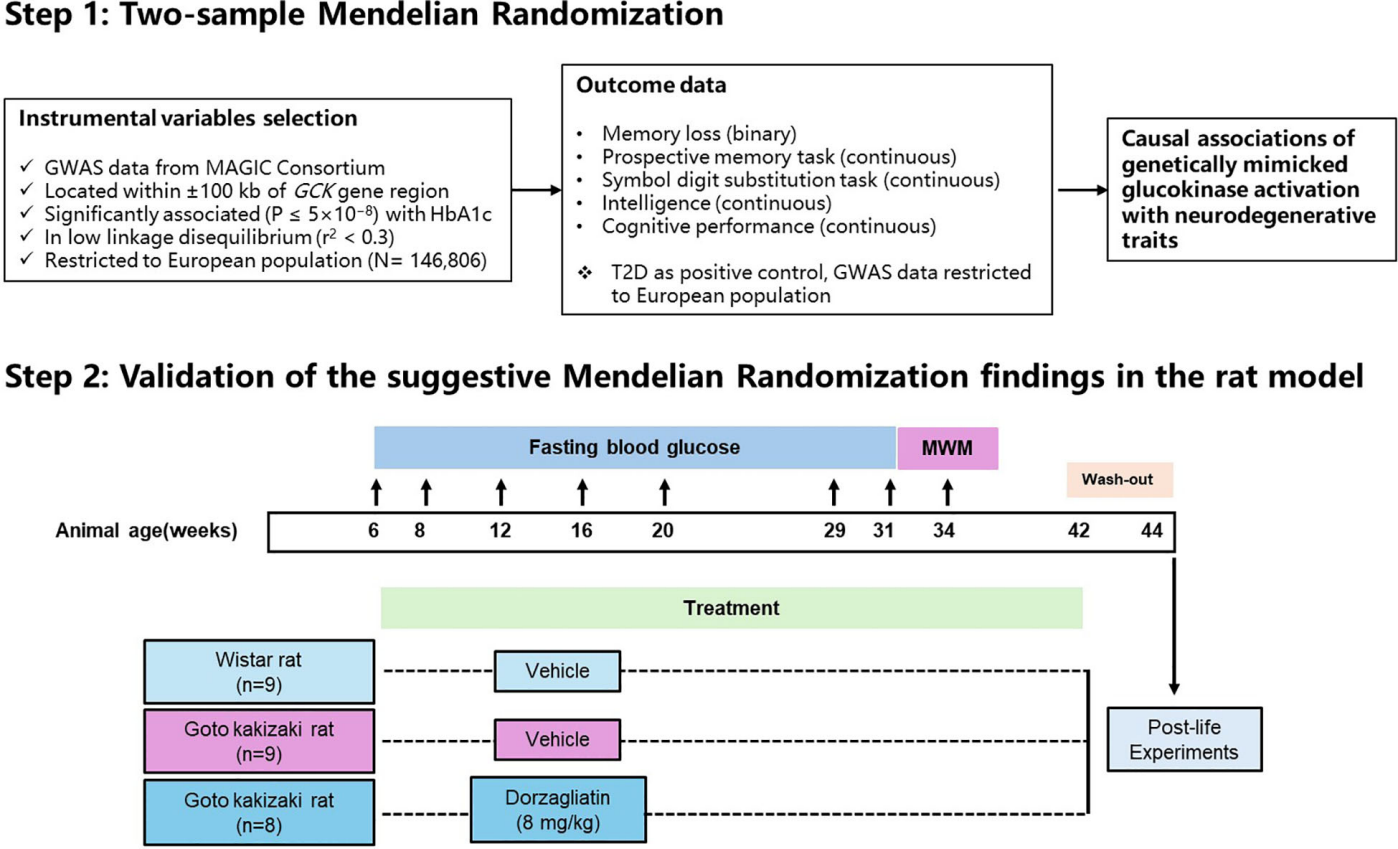
Flowchart of the study design.

For the first part of our study, we carried out a two-sample MR analysis using publicly accessible summary statistics obtained from genome-wide association studies (GWAS). We investigated causal associations of genetically mimicked GK activation with neurodegenerative traits. For the second part of the study, we validated the suggestive MR findings in the Goto Kakizaki rat model and explored the potential underlying mechanisms. **Figure 1** shows the flowchart of the study design.

### Two-sample MR study

#### Instrumental variables selection

To choose instrumental variables (IVs) that mimic GK (gene symbol, *GCK*) activation, we identified single nucleotide polymorphisms (SNPs) located within 100 kb downstream and upstream of *GCK* gene region (genomic position chromosome 7: 44182812–44229038 on build GRCh37.p13). These variants should exhibit a strong correlation (P ≤ 5×10^−8^) with HbA1c (unit, %) in the summary statistics of Meta-Analyses of Glucose and Insulin-related traits Consortium (MAGIC) study (17). SNPs were filtered by linkage disequilibrium clumping algorithm in the PLINK (window size=10000KB, r^2^ threshold=0.3, the 1000 Genomes European Project as the reference panel) and those with the strongest significance were retained. The exposure data for IVs selection were restricted to the European population (N = 146,806) to avoid potential bias from population difference.

#### Outcome data sources

We evaluated the impact of genetically mimicked GK activation on cognitive related traits including memory loss (binary), prospective memory task (continuous), symbol digit substitution task (continuous), intelligence (continuous) and cognitive performance (continuous), as defined by the original studies. In addition, we used T2D as positive control since that GKA is a class of antidiabetic agents.

**Supplementary Table S1** summarizes the public data used for outcomes. These summary statistics were all derived from the subjects of European ancestry and the populations did not overlap with those in the MAGIC study (exposure data). Briefly, summary statistics for memory loss were obtained from the FinnGen database (1,384 European cases and 210,333 European controls, https://r5.finngen.fi/pheno/MEMLOSS). Summary statistics for the scores of prospective memory task (sample size 162,335 Europeans), symbol digit substitution task (sample size 84,125 Europeans) and intelligence (sample size 216,381 Europeans) were obtained from a GWAS published by Hatoum AS et al. (18). Summary statistics for cognitive performance were obtained from a GWAS published by Lee JJ et al. (sample size 257,841 Europeans) (19). Summary statistics for T2D were obtained from a GWAS published by Sakaue S et al. (38,841 European cases and 451,248 European controls) (20). More detailed definitions and diagnoses of the traits can be found in the respective original publications or consortium websites.

#### Comparing effects of glucokinase activation with general HbA1c lowering

To assess if the observed MR results for genetically mimicked GK activation were only a result of HbA1c improvement, we also evaluated the causal impact of genetically predicted lower HbA1c on outcomes of interest. We identified genetic variants in the MAGIC study associated with HbA1c throughout the genome (exclude *GCK* gene region) to mimic general HbA1c lowering (European ancestry, P ≤ 5×10^−8^, window size=10000KB, r^2^ <0.001, the 1000 Genomes European Project as the reference panel).

### Animal study

All *in vivo* experiments were conducted at WuXi AppTec Co., Ltd. and animal procedures were approved by the Institutional Committee Animal Care and Use Committee of WuXi AppTec Co., Ltd (Approval No. GP02-047-2020v1.0, Approval Date 20^th^ March 2020.). The biochemistry experiments were conducted at the Interdisciplinary Research Center on Biology and Chemistry, Shanghai Institute of Organic Chemistry, Chinese Academy of Sciences, Shanghai, China.

#### Animal models

Male Goto Kakizaki rats (4–5 weeks) and age-matched male Wistar rats were used in the present study. All the animals in this study received proper animal care in compliance with the institutional regulations. These rats were maintained in cages (2 rats per cage) under a 12-hour light/dark cycle (light phase between 7:00-19:00) with temperature at 23 ± 2°C and humidity at 50 ± 5%. The rats were permitted free access to food and water. After one week of adaptation, 17 Goto Kakizaki rats and 9 Wistar rats with proper baseline fasting blood glucose (FBG) and weight were selected for the treatment, and the Goto Kakizaki rats were randomly allocated into vehicle group and dorzagliatin group. Throughout the 36-week treatment period, both the Goto Kakizaki and Wistar rats in vehicle group were intragastrically administered twice daily with a vehicle composed of 0.5% hydroxypropyl cellulose-L and 0.1% Tween 80. In parallel, the Goto Kakizaki rats in dorzagliatin group received 8 mg/kg dorzagliatin. Fasting blood glucose (FBG) levels were measured at the following time points (rat age): 6-week-old (baseline), 8-, 12-, 16-, 20-, 29-, and 31-week-old. The Morris water maze (MWM) test was conducted when rats were 34-week-old (after 28 weeks of continuous administration). To distinguish between acute pharmacological effects and sustained adaptations induced by chronic treatment, a 2-week wash-out period was observed after the whole 36-week treatment (at age of 42-week-old). At the age of 44 weeks, all animals were euthanized using compressed carbon dioxide (CO_2_, ≥95%) at a controlled flow rate of 8.1–19 L/min (30%-70% of the chamber volume per minute), with death confirmed by cervical dislocation, and tissue samples were collected for biochemistry experiments and pathology experiments. A detailed schematic timeline is provided in **Figure 1**.

#### Morris water maze test

The spatial learning and memory performance of all rats were evaluated using the MWM test after receiving dorzagliatin or vehicle treatments. A pool with a diameter of 135 cm and depth of 45 cm was filled with opaque water at 22°C. The pool was then divided into four equivalent virtual quadrants (northeast, southeast, southwest and northwest).

A platform was submerged in one of the quadrants which was defined as the target quadrant, and the position of the hidden platform remained unchanged during the training. Prior to the commencement of each daily experiment, the rats were moved to a preparation room where they were given a minimum of 30 minutes to acclimatize their environment. During the place navigation training session, the rats were randomly placed into the water in any of the four quadrants facing the wall. If the rats found the platform within 60 seconds, they were allowed to remain on the platform for 15 seconds. The time to locate the platform (escape latency) was recorded. For those could not find the platform or failed to escape within 60 seconds were gently placed on the platform for 15 seconds and the escape latency was recorded as 60 seconds. All rats were trained 4 times a day (30 minutes apart) for 5 days. The platform was removed on day 6 of the spatial probe test and the rats were placed into the water at the same location. The ANY-maze video tracking system was used to record the escape delay, number of entries, dwell time, and swimming distance in the target quadrant.

#### Western blot analysis

Western blot analyses were performed by standard protocol and protein levels of hippocampal N-methyl-D-aspartate (NMDA) receptor subunits (GluN1, GluN2A), glucose transporters (GLUT1 and GLUT3), insulin receptors (IR-A and IR-B) and postsynaptic density protein-95 (PSD95) were measured. Briefly, hippocampus samples were collected (n=4/group) and homogenized in RIPA lysis buffer (Solarbio, Cat. No: R0010). The protein concentration was determined using a bicinchoninic acid (BCA) protein assay kit (Thermo Scientific, Cat. No: 23227). Samples were electrophoresed on 8-12% sodium dodecyl sulfate (SDS)-polyacrylamide gels which were then transferred to the polyvinylidene difluoride (PVDF) membranes (tank blotting for GluN1 and GluN2A; Semi-dry blotting for GLUT1, GLUT3, IR and PSD95). The membranes were blocked with 1×TBST and 5% BSA (tank blotting) or 5% skim milk (Semi-dry blotting) for 1 hour at room temperature and then incubated overnight at 4°C with following primary antibodies, respectively: mouse monoclonal anti-GluN1 antibody (Millipore, Cat. No: 05-432); rabbit polyclonal anti-GluN2A antibody (NOVUS, Cat. No: NB300-105); rabbit monoclonal anti-GLUT1 antibody (Abcam, Cat. No: ab115730); rabbit polyclonal anti-GLUT3 antibody (Bioss, Cat. No: bs-1207R); rabbit polyclonal anti-IR antibody (Abcam, Cat. No: ab137747); rabbit monoclonal anti-PSD95 antibody (Abcam, Cat. No: ab238135). Following incubation overnight at 4˚C with primary antibodies, the membranes were washed with TBST three times for 10 min and were then incubated with the corresponding secondary antibodies (Goat Anti-Rabbit IgG, YESEN, 33101ES60 or Goat Anti-Mouse IgG, YESEN, 33201ES60) for 1.5 hours at room temperature. Glyceraldehyde-3-phosphate dehydrogenase (GAPDH, Proteintech, Cat. No: 60004–1) and β-actin (Cell Signaling Technology, Cat. No:3700S) were used as the loading control. The signal was detected using the enhanced chemiluminescence (ECL) detection reagent kit (Yeasen Biotechnology, Cat. No: 36208ES60) and E-blot Touch Imager (e-BLOT Life Science). The densitometry was measured using ImageJ.

### Statistical analysis

In the two-sample MR study, we determined the HbA1c-decreasing allele as the effect allele based on the anticipated impacts of GK activation in the MAGIC study. The genetic associations of IVs with the exposures and outcomes were then harmonized by aligning the effect alleles. We calculated the F-statistic to ensure the robustness of IVs used in the MR analyses (21). The Wald ratio (the ratio of the genetic association with outcome to the genetic association of exposure) for each SNP was calculated (22). We combined the Wald ratios using the random-effects inverse variance-weighted (IVW) method to estimate the causal effects of the exposure on the outcomes (23). Different sensitivity method were used to detect the heterogeneity (Cochran’s Q statistic) (24), horizontal pleiotropy (MR-Egger regression) (25), and the potential outliers (MR-PRESSO method) (26). All MR analyses were performed using R software (version 4.3.2) with packages “TwoSampleMR” and “MRPRESSO”. A P-value less than 0.05 was regarded statistically significant.

Unless otherwise noted in the figure legends, the statistical significance in animal study is determined by unpaired, two-tailed Student’s t-test. Error bars represent the standard error of the mean (SEM).

## Results

### Causal effects of genetically mimicked glucokinase activation

**Figure 2:**
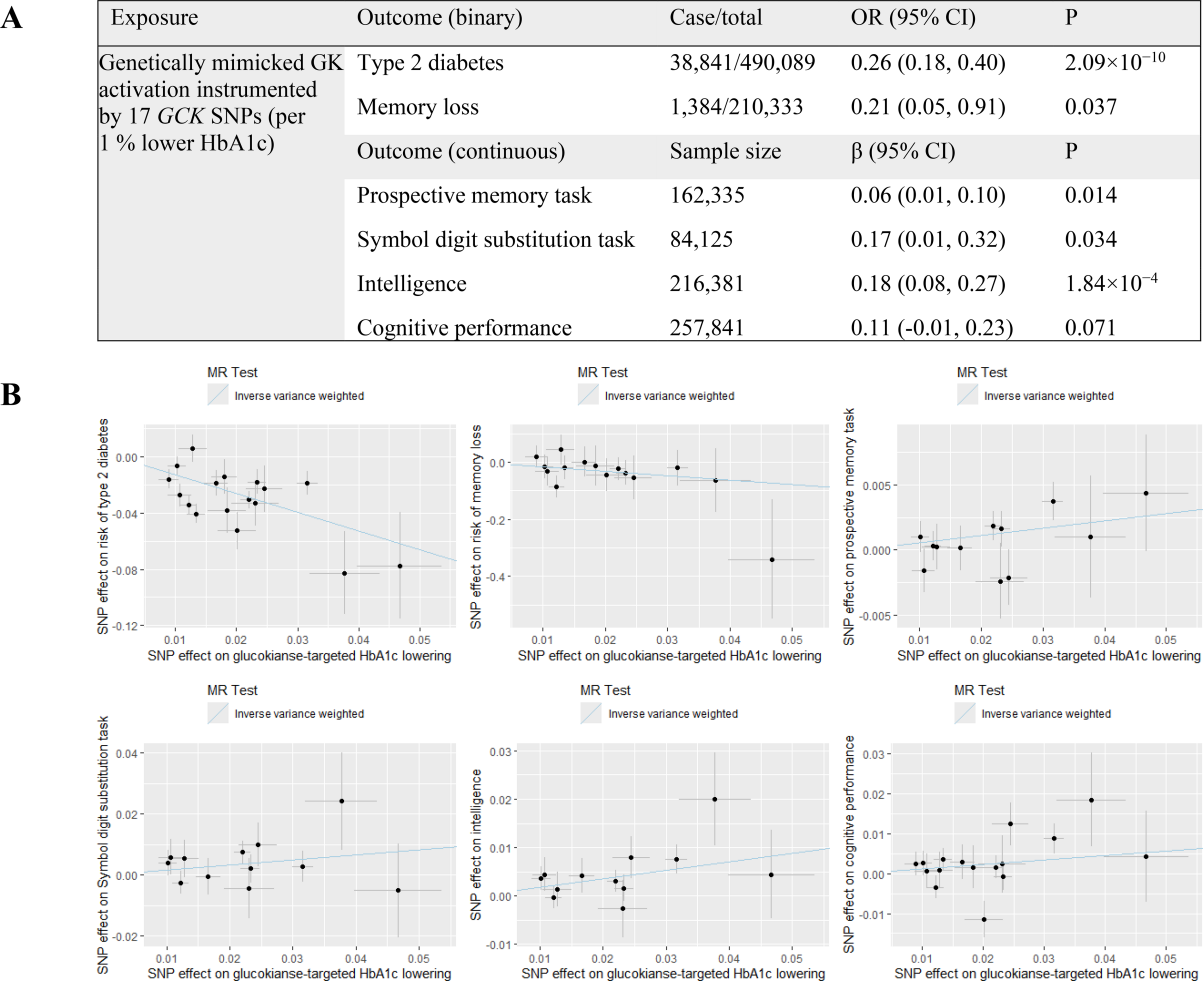
Causal effects of genetically mimicked GK activation on the outcomes. **(A)** Causal estimates derived from two-sample MR analyses. The population was restricted to European ancestry. Estimations were based on the inverse variance-weighted method; **(B)** Scatter plot of SNP effect on glucokinase-targeted glucose lowering versus outcomes of interest. GK, glucokinase; SNP, single nucleotide polymorphism; OR, odds ratio; CI, confidence interval; MR, Mendelian randomization.

Seventeen SNPs (**Supplementary Table S2**) located in or around *GCK* gene associated with HbA1c (%) were identified from the MAGIC study as IVs (F-statistic =94) to mimic the activation of GK. For positive control, genetically mimicked GK activation was associated with reduced risk of T2D (OR 0.26 per 1% lower HbA1c, 95% CI 0.18 to 0.40, P = 2.09×10^−10^) (**Figure 2**). These results confirm the validity of the IVs selection. We found that genetically mimicked GK activation resulted in a decreased risk of memory loss (OR 0.21 per 1% lower HbA1c, 95% CI 0.05 to 0.91, P = 0.037), and was associated with higher scores of prospective memory task (β 0.06 per 1% lower HbA1c, 95% CI 0.01 to 0.10, P = 0.014), symbol digit substitution task (β 0.17 per 1% lower HbA1c, 95% CI 0.01 to 0.32, P = 0.034) and intelligence (β 0.18 per 1% lower HbA1c, 95% CI 0.08 to 0.27, P = 1.84×10^−4^) (**Figure 2**). There was no significant heterogeneity in the IVW model (P_Q-statistic_ =0.915 for memory loss, 0.634 for prospective memory task, 0.759 for symbol digit substitution task and 0.710 for intelligence) nor horizontal pleiotropy in MR-Egger regression test (P_egger-intercept_ =0.841 for memory loss, 0.329 for prospective memory task, 0.808 for symbol digit substitution task and 0.848 for intelligence) (**Supplementary Table S3**). No outliers were detected in the MR-PRESSO model (P_global-test_ =0.926 for memory loss, 0.609 for prospective memory task, 0.759 for symbol digit substitution task and 0.737 for intelligence). The causal associations for cognitive performance score showed a tendency toward improvement, albeit not significant (β 0.11 per 1% lower HbA1c, 95% CI -0.01 to 0.23, P = 0.071) (**Figure 2**).

### Causal effects of genetically mimicked general glycemic control

**Figure 3:**
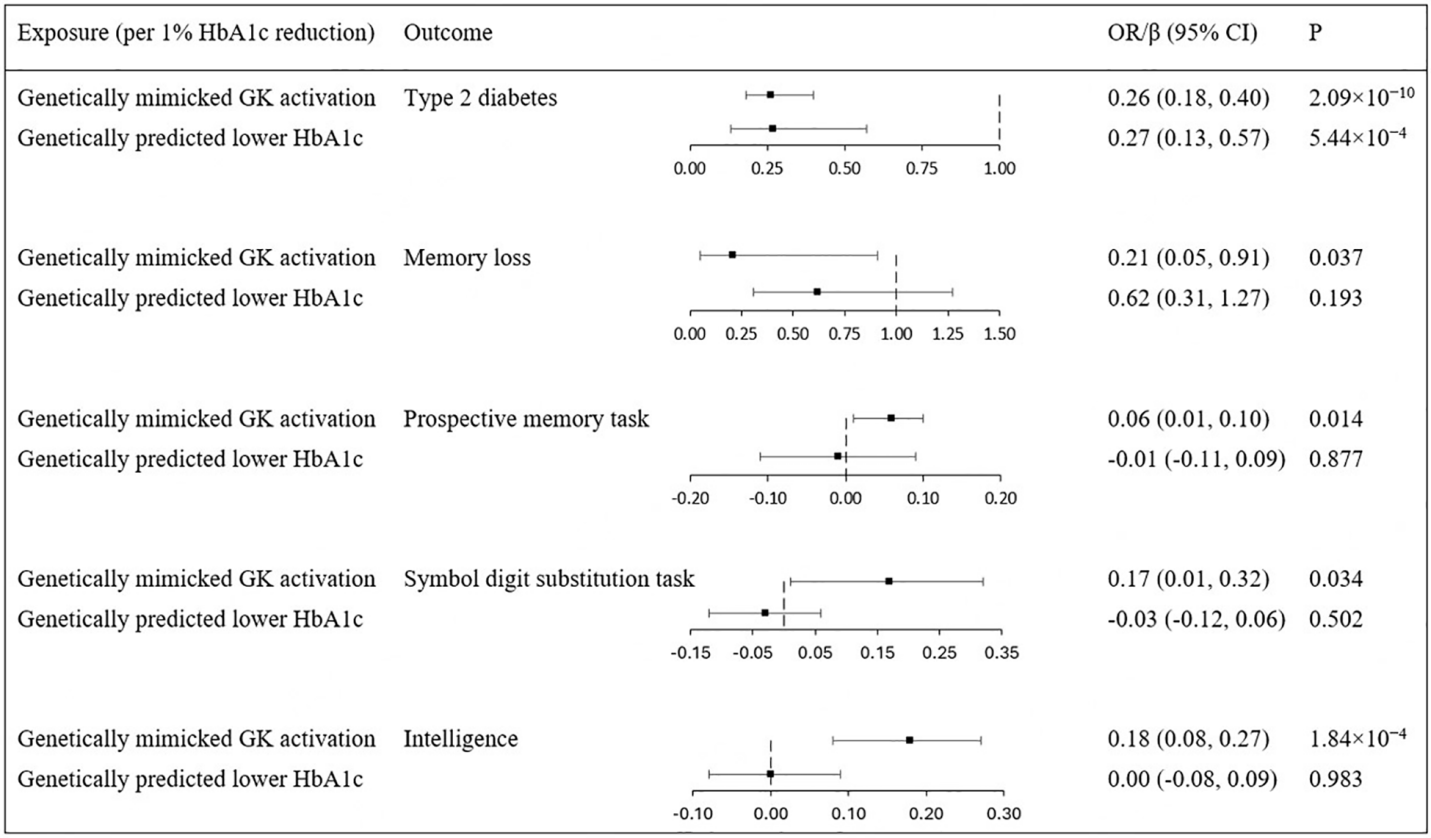
Effects of genetically mimicked GK activation versus genetically predicted lower HbA1c. All estimations were based on the inverse variance weighted method. The population was restricted to European ancestry. GK, glucokinase; OR, odds ratio; CI, confidence interval.

We have additionally identified 73 genome-wide SNPs (**Supplementary Table S4**) (F-statistic =103) to mimic general glycemic control in terms of HbA1c reduction. As shown in **Figure 3**, genetically predicted lower HbA1c significantly reduced the risk of T2D (OR 0.27 per 1% lower HbA1c, 95% CI 0.13 to 0.57, P = 5.44×10^−4^) to a similar degree as genetically mimicked GK activation. However, genetically predicted lower HbA1c was not associated with the risk of memory loss (OR 0.62 per 1% lower HbA1c, 95% CI 0.31 to 1.27, P = 0.193). Genetically predicted HbA1c lowering also did not influence the scores of prospective memory task (β -0.01 per 1% lower HbA1c, 95% CI -0.11 to 0.09, P = 0.877), symbol digit substitution task (β -0.03 per 1% lower HbA1c, 95% CI -0.12 to 0.06, P = 0.502) nor intelligence (0.983 β 0.00 per 1% lower HbA1c, 95% CI -0.08 to 0.09, P = 0.983). Thus, the observed cognition-related protective effects associated with GK activation may not be simply attributed to glycemic lowering effects. Maintaining glucose homeostasis through GK activation may offer direct impact of brain functions and disease prevention.

### Dorzagliatin maintained blood glucose levels and protected against spatial memory deficit in Goto Kakizaki rats

**Figure 4:**
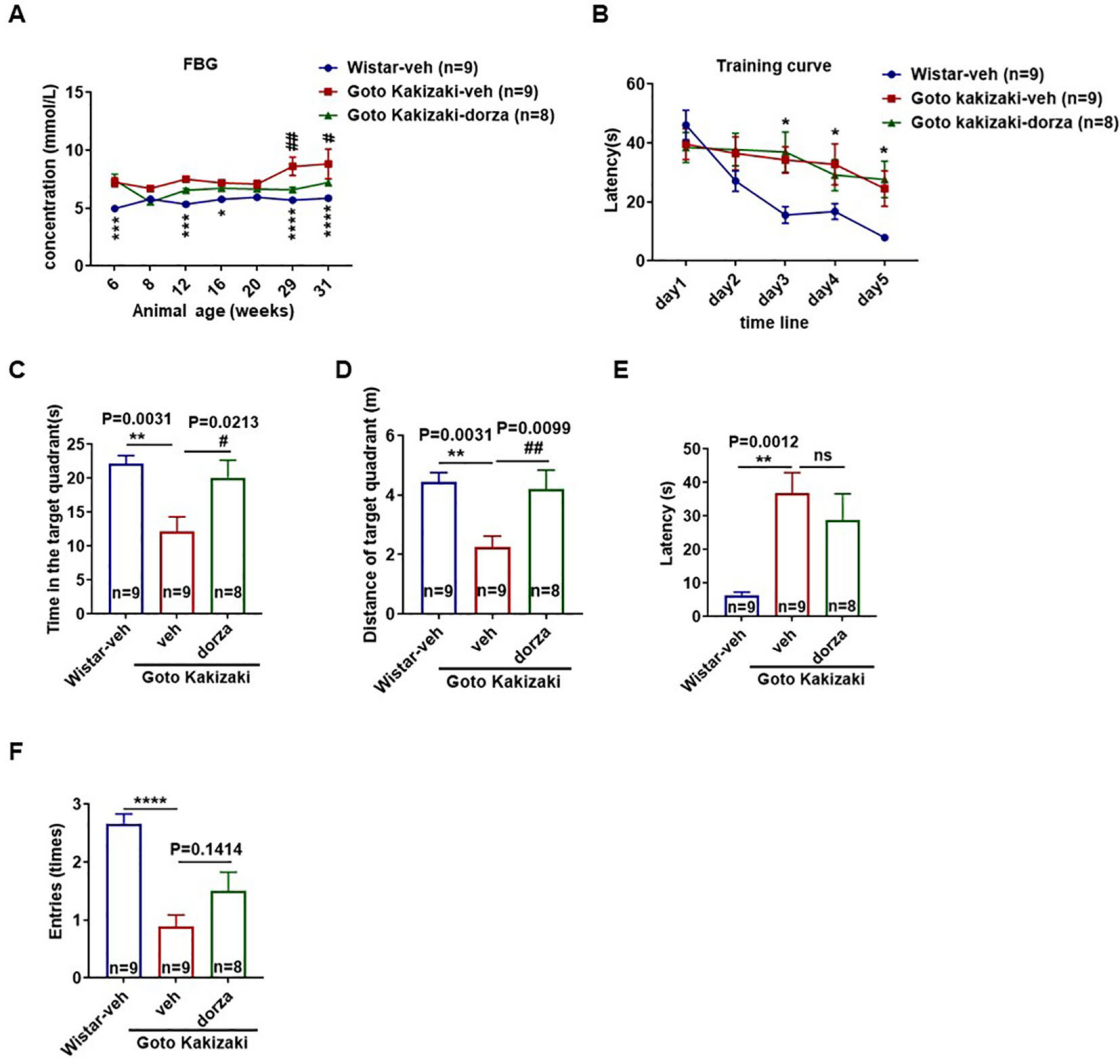
Maintenance of glucose homeostasis protects against diabetes-induced memory impairment. **(A)** The fasting blood glucose level in each group. **(B)** Training curves during the acquisition phase of the Morris water maze test. **(C, D)** Time spent and total distance in target quadrant by each group of animals during probe tests. **(E, F)** Latency to first entry to target quadrant **(E)** and total number of entries to the target **(F)** during probe trial. Numbers of animals measured for each condition are indicated in the individual bars. The data are expressed as mean ± SEM; Two-way ANOVA was used for comparisons in **(A, B)** One-way ANOVA with multiple comparison test for C-F. Goto kakizaki-vehicle compared with Wistar-vehicle group, *P<0.05, **P<0.01, ***P<0.001, ****P<0.0001. Goto kakizaki-vehicle compared with Goto kakizaki-dorzagliatin group, #P<0.05, ##P<0.01. veh, vehicle; dorza, dorzagliatin.

We then conducted animal studies to provide experimental evidence for our MR findings. In the Goto Kakizaki-vehicle group, the fasting blood glucose (FBG) levels were increased at older ages and were significantly higher than those in the vehicle treated Wistar group, suggesting a disruption of glucose homeostasis in aged Goto Kakizaki rats (**Figure 4A**). Continuous administration of low-dose dorzagliatin ameliorated the FBG elevation in the Goto Kakizaki rats compared with the vehicle treatment and was similar to the FBG levels observed in age-matched Wistar-vehicle rats (**Figure 4A**).

We utilized MWM test to assess alterations in spatial learning and memory in rats following 28 weeks (at age of week 34) of continuous administration of dorzagliatin or vehicle. Based on the learning curves of the place navigation test (**Figure 4B**), the escape latency was decreased following training for all groups. Among these groups, the Wistar-vehicle rats showed the best learning performance. The administration of dorzagliatin did not improve the learning ability of Goto Kakizaki rats (**Figure 4B**). Following the removal of the platform in the spatial probe test, the time spent and traveled distance in the target quadrant were significantly lower in the Goto Kakizaki-vehicle group compared with dorzagliatin treated Goto Kakizaki rats and Wistar-vehicle group (**Figures 4C, D**). Further, the latency and number of entries to the target quadrant for 60 seconds were also measured (**Figures 4E, F**). The Goto Kakizaki-vehicle rats exhibited poorer memory, as evidenced by significantly longer escape latencies and fewer times of entries compared with the Wistar-vehicle rats (**Figures 4E, F**). Dorzagliatin fed Goto Kakizaki rats showed better memory compared to the rats in Goto Kakizaki-vehicle group. They spent more time and traveled longer in the target quadrant (**Figures 4C, D**), and they were able to reach the target quadrant in less time with more entries, although the differences were nonsignificant (**Figures 4E, F**). These data suggested that continuous administration of low-dose dorzagliatin protected against the developmental hyperglycemia and may have a protective effect against memory loss in Goto Kakizaki rats.

### Dorzagliatin showed protective effects against glucose metabolic alteration in the brain

**Figure 5:**
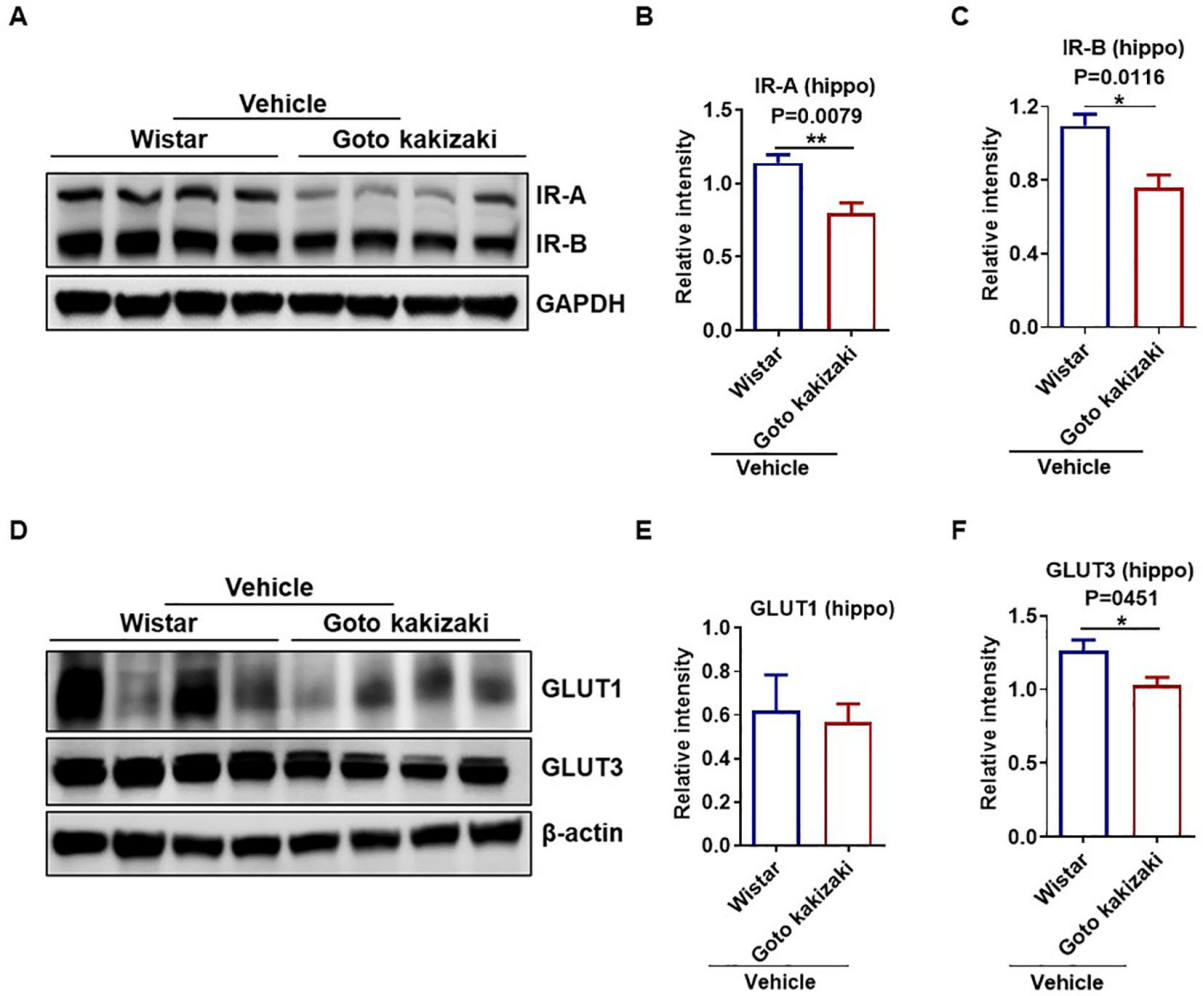
Dysregulation of insulin receptors and glucose transporters in the hippocampus of Goto Kakizaki rats. **(A–C)** Western blot and protein quantification of two insulin receptor isoforms (IR-A and IR-B) in the hippocampus of Goto kakizaki-vehicle and Wistar-vehicle rats. **(D–F)** Representative Western blot and protein quantification of two glucose transporters (GLUT1 and GLUT3) in hippocampus of Goto kakizaki-vehicle and Wistar-vehicle rats. All values are presented as the mean ± SEM. Student’s t test, two tailed. *P < 0.05, **P < 0.01. Protein levels were normalized relative to the β-actin or GAPDH loading control, n=4 per group; hippo, hippocampus.

**Figure 6:**
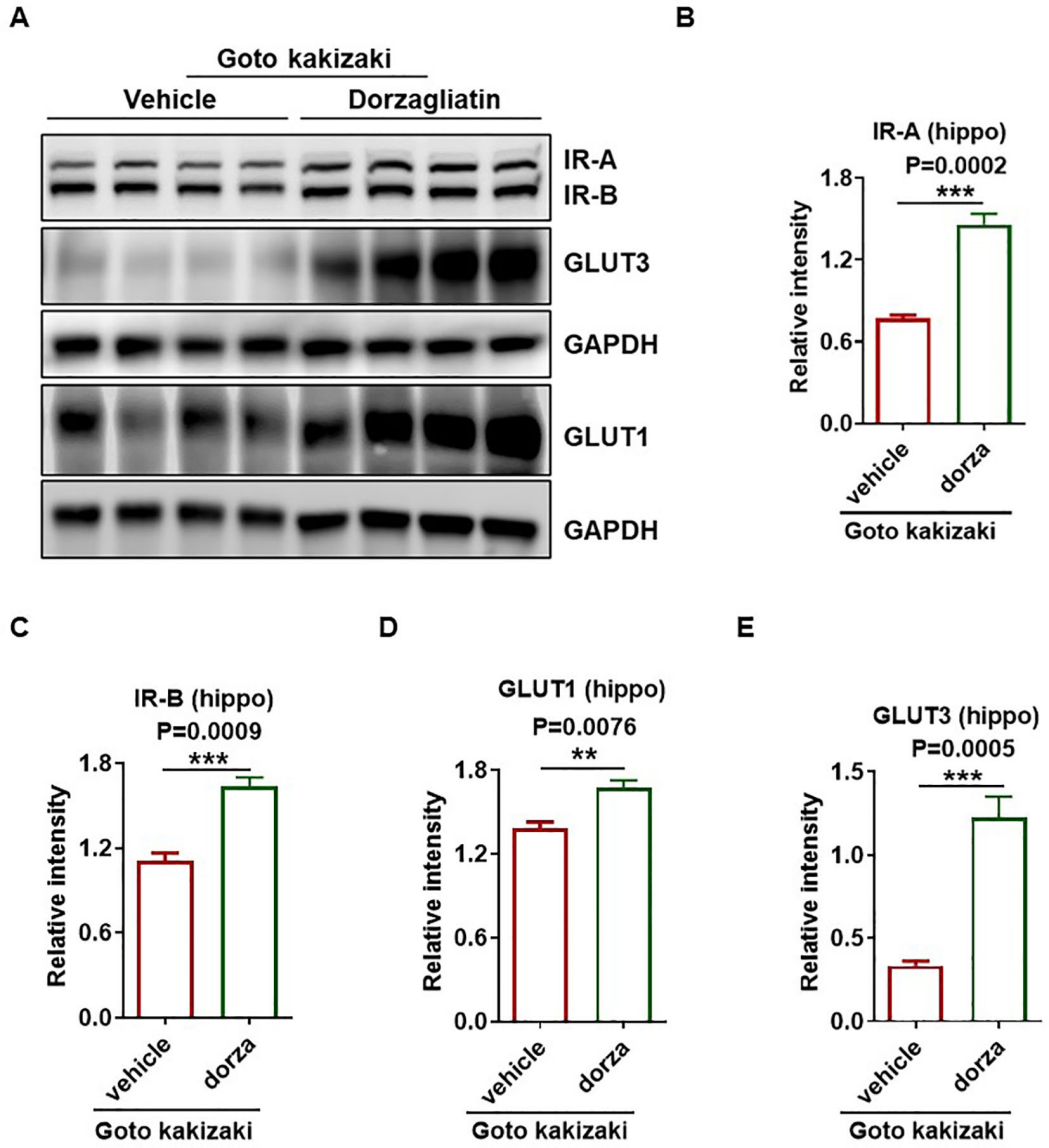
Dorzagliatin prevented downregulation of insulin receptors and glucose transporters in the hippocampus of Goto Kakizaki rats. **(A)** Western blot of two insulin receptor isoforms (IR-A and IR-B) and glucose transporters (GLUT1 and GLUT3) in the Goto Kakizaki rat hippocampus after treatment of vehicle or dorzagliatin. **(B, C)** Statistics of IR-A and IR-B protein levels in the hippocampus of vehicle or dorzagliatin treated Goto Kakizaki rats. **(D, E)** Quantification of GLUT1 **(D)** and GLUT3 **(E)** protein levels in the hippocampus of each group. All values are presented as the mean ± SEM. Student’s t test, two tailed. **P < 0.01, ***P < 0.001. Protein levels were normalized relative to the β-actin or GAPDH loading control, n=4 per group; hippo, hippocampus; dorza, dorzagliatin.

To further elucidate the mechanism of memory impairment in Goto Kakizaki rats and the protective role of dorzagliatin, we measured the protein levels of insulin receptors and glucose transporters specifically in the hippocampus. As shown in **Figure 5**, insulin receptors (IR-A and IR-B) and GLUT3 levels were significantly decreased in Goto Kakizaki-vehicle group compared with the Wistar-vehicle group (**Figures 5A–D, F**). The GLUT1 level in Goto Kakizaki-vehicle rats also showed a slight reduction although not significant (**Figures 5D, E**). In contrast, the protein expression levels of insulin receptors and glucose transporters significantly increased in dorzagliatin treated rats compared with those in the Goto Kakizaki -vehicle group (**Figures 6A–E**). These data suggested that peripheral hyperglycemia disrupted systemic glucose homeostasis, leading to metabolic alterations in the hippocampus of Goto Kakizaki rats through reduced glucose uptake and insulin sensitivity in the brain. The administration of low-dose dorzagliatin to the peripheral region also effectively attenuated the development of insulin resistance and metabolic disturbance in the brain.

### Dorzagliatin stabilized expression of synapse-associated proteins in the hippocampus

**Figure 7:**
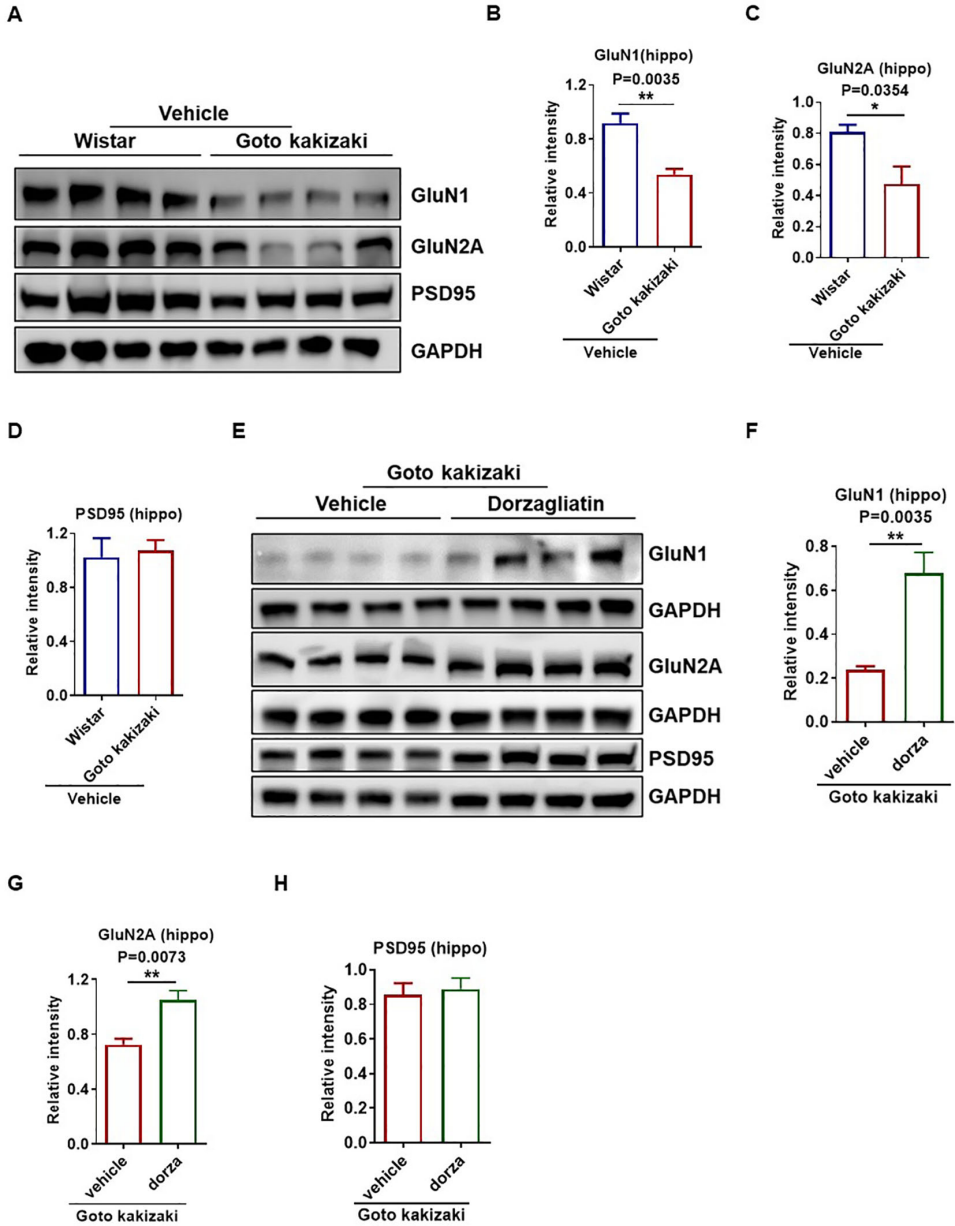
Dorzagliatin prevented diabetes-induced downregulation of synaptic proteins in Goto Kakizaki rats. **(A)** Western blot analysis of selected glutamate receptors and postsynaptic density protein 95 (PSD-95) in hippocampus of each group. GAPDH was used as an internal control. **(B–D)** Statistics of GluN1 **(B)**, GluN2A **(C)** and PSD-95 **(D)** protein levels in the hippocampus of T2D (Goto Kakizaki-vehicle) and control (Wistar-vehicle). **(E)** Western blot analysis of selected glutamate receptors and postsynaptic density protein 95 (PSD-95) in the hippocampus of Goto Kakizaki-vehicle and Goto Kakizaki-dorzagliatin rats. **(F–H)** Statistics of GluN1 **(F)**, GluN2A **(G)** and PSD-95 **(H)** protein levels in the hippocampus of Goto Kakizaki-vehicle group and Goto Kakizaki-dorzagliatin group. Data are expressed as mean ± SEM. Student’s t test, two tailed. *P < 0.05, **P < 0.01. (n=4 per group). hippo, hippocampus; dorza, dorzagliatin.

To investigate the mechanisms underlying by which dorzagliatin ameliorated memory deficits in Goto Kakizaki rats beyond the glucose lowering effect, we examined the expression levels of several synaptic proteins. We observed a significant decrease in the levels of GluN1 and GluN2A in the hippocampus of Goto Kakizaki-vehicle rats compared to Wistar rats (**Figures 7A–C**). However, there was no difference in PSD95 level between the two groups (**Figures 7A, D**). Continuous administration of low-dose dorzagliatin effectively prevented the down-regulation of synapse-associated proteins (GluN1 and GluN2A) in the hippocampus of Goto Kakizaki rats (**Figures 7E–G**). Additionally, the levels of PSD95 remained unchanged (**Figures 7E, H**). These findings suggested that glucose homeostasis through GK activation played an important role in stabilizing synaptic protein levels, providing insights into the molecular mechanisms behind the protective effects of dorzagliatin on memory.

## Discussion

Our study demonstrated that genetic activation of GK correlated with reduced risk of memory loss and with improved intelligence through Mendelian randomization study. The effects outperformed HbA1c reduction through other means, suggesting additional mechanism related to GK regulated pathway, especially glucose homeostasis. In the Goto Kakizaki rat study, low-dose GKA dorzagliatin protected the defect of spatial memory and reduction of critical glucose transmitter GLUT1 and GLUT3 expression, as well as protected insulin receptor expression in the brain. The additional finding is that dorzagliatin treatment improved the impaired expression of ion gated glutamate receptor GluN2A and GluN1 in the synapse of neuron transduction. Through glucose-lowering-dependent mechanism, dorzagliatin could reduce chronic hyperglycemia-induced neurotoxicity and improve insulin signaling in the brain; through glucose-lowering-independent mechanism, dorzagliatin could maintain energy supply and promote synaptic plasticity and neurogenesis. The multiple benefits to the brain function are not a direct effect of dorzagliatin which does not pass through blood-brain barrier (no radioactive distribution was detected in the central nervous system following a single oral administration of [_14_C]-dorzagliatin at 50 mg/kg, unpublished data). We believe that the effect of dorzagliatin in the restoration of glucose homeostasis provides the foundation for the prevention of neurodegeneration related diseases. Glucose homeostasis is fundamental to maintain energy supply in highly glucose-dependent tissues such as the brain and red blood cells (27). The energy demand of brain is required to restore the ion gradients in electrical signal transmission and for the biochemical neurotransmitter formation, uptake and recycling. In this line, when ATP production does not fulfill energy requirements (situations such as aging, ischemia and/or neurodegenerative disorders), synapses appear to be more vulnerable to dysfunction and degeneration. Within the central nervous system, disrupted glucose metabolism is a hallmark of neurodegenerative diseases, including Alzheimer’s disease (28), Parkinson’s disease (29, 30) and Huntington’s disease (31). In particular, DACD represents a common yet underrecognized complication of diabetes for which effective therapeutic interventions are urgently needed (32–34).

Glucokinase activators, such as dorzagliatin, represent a novel class of oral anti-diabetic drugs that enhance glucose homeostasis by improving insulin and GLP-1 secretion, reducing insulin resistance, and improving β-cell function (35–37), providing new hope for treatment of DACD. A recent clinical trial demonstrated that dorzagliatin, in combination with metformin improved Montreal Cognitive Assessment (MoCA) scores (a key cognitive function indicator) and glycemic control in T2D patients with MCI, outperforming metformin monotherapy. These findings suggest that dorzagliatin may offer therapeutic value beyond glycemic control, providing a new potential direction for treating T2D-associated cognitive dysfunction (38). In this study, we used two-sample MR to investigate the lifelong effects of GK activation on cognitive traits. Genetic proxies for GK activation within the *GCK* gene were associated with a reduced risk of memory loss and improved performance in prospective memory, executive function, and intelligence. Importantly, these effects were not replicated using genetic instruments for general glycemic control, indicating that GK activation may support cognition through mechanisms independent of glucose lowering. Consistent with MR findings, animal studies in Goto Kakizaki rats showed that low-dose (8 mg/kg) dorzagliatin administered from a young age prevented hyperglycemia and cognitive decline in the Morris water maze test. The dose of dorzagliatin (8 mg/kg) used in this study was selected based on a previously reported minimum effective dose in diabetic rodents (10 mg/kg) (39), and was adjusted downward for chronic, early intervention in the young, prediabetic rat model.

There is substantial evidence suggesting a connection between altered insulin action and dysregulated glucose metabolism in both animal models and individuals with dementia (40, 41). Insulin signaling modulates behavioral responses and metabolic processes by governing the uptake of glucose into the brain through glucose transporters, thereby maintaining systemic glucose homeostasis (42). Recent studies indicated that the absence of insulin receptors in the brain led to metabolic and behavioral changes, including reduced glutamate receptor and impaired cognitive performance in rodents (43, 44). This is significant as similar reductions in insulin and its receptors have been observed in the brains of Alzheimer’s disease patients. Some researchers posit that insulin resistance may trigger the formation of amyloid plaques, inflammation, and oxidative stress, which are hallmarks of cognitive decline (45). Furthermore, impairment in insulin signaling, such as insulin resistance, may accelerate brain aging, potentially impacting synaptic plasticity and contributing to neurodegeneration (43, 44). This study found that the insulin receptors in the hippocampus of vehicle-treated Goto Kakizaki rats were significantly reduced compared with the Wistar rats, and treatment of dorzagliatin prevented this downward trend. A similar change pattern was also observed in the vehicle or dorzagliatin-treated rats for expression levels of key glucose transporters (GLUT1 and GLUT3), which are responsible for glucose uptake to enable energy supply in the brain. GLUT1 mediates the uptake of glucose from the extracellular fluid by astrocytes, oligodendrocytes, and microglia, while GLUT3 promotes the uptake of glucose by neurons. GLUT3 couples with hexokinase 1 converts glucose into glucose-6-phosphate which leads to the synthesis of ATP and neurotransmitters of glutamate and γ-aminobutyric acid (GABA). Reduced expression of glucose transporters in neurons and glial cells might result in decreased neuronal glucose transport and an insufficient cerebral glucose supply, thereby contributing to the mechanisms of impaired memory (46, 47). Patients with Alzheimer’s disease also exhibit lower levels of brain GLUT1 and GLUT3 (48, 49), and the reductions of these transporters have been related with tau hyperphosphorylation (50). The preventive effects of dorzagliatin on downregulation of expression levels of insulin receptors and glucose transporters implied its protective effects on DACD via regulating disordered metabolic function and maintaining proper glucose homeostasis in the brain.

Moreover, dorzagliatin prevented the reduction in NMDA receptor subunits (GluN1 and GluN2A) which are another important class of synaptic proteins involved in cognitive, motor and psychiatric function via the mediation of glutamatergic synaptic transmission throughout the central nervous system (51, 52). It has been suggested that insulin in the brain may enhance neurite outgrowth and boost synaptic functionality and plasticity (53, 54). Since glucose is a fundamental precursor for glutamate and GABA synthesis, adequate glucose supply through functional transporters is essential for maintaining neurotransmitter balance and synaptic plasticity. Our data imply that dorzagliatin may break the vicious cycle of energy failure and neurodegeneration by maintaining glucose transporter expression and insulin signaling, thereby supporting neuronal energy supply and synaptic function, as shown in **Supplementary Figure S1**.

We acknowledge certain limitations in our study. The effect sizes (OR and β values) in the MR study reflected effects of each unit HbA1c reduction (%) caused by GK activation but cannot be directly utilized to extrapolate the clinical effects of GKA. This is because RCTs only investigate the short-term effects of pharmacological treatment as opposed to the impacts of lifelong exposure estimated by MR (55). Moreover, our MR analysis was restricted to European populations due to very limited data in Asians; future MR validation in Asian population is warranted when such GWAS datasets are available. Although animal results support the genetic findings, cognitive assessment was limited to spatial memory; future studies should include additional domains such as working and fear memory.

## Conclusions

Integrating genetic and experimental evidence, we demonstrate that GK activation by dorzagliatin may possess additional neuroprotective effects against diabetes-induced cognitive impairment that extend beyond those achieved through general glycemic control, possibly due to the maintenance of cerebral glucose homeostasis via preservation of glucose transporters (GLUT1 and GLUT3), enhanced insulin signaling, and support of NMDA receptor-mediated synaptic transmission. Collectively, our findings position dorzagliatin as a promising therapeutic candidate for preventing or treating DACD, warranting confirmation in future definitive clinical trials.

## Data Availability

The original contributions presented in the study are included in the article/**Supplementary Material**. Further inquiries can be directed to the corresponding authors.

## References

[1] Jayaraman A Pike CJ . Alzheimer’s disease and type 2 diabetes: multiple mechanisms contribute to interactions. Curr Diabetes Rep. (2014) 14:476. doi: 10.1007/s11892-014-0476-2, PMID: 24526623 PMC3985543

[2] You Y Liu Z Chen Y Xu Y Qin J Guo S . The prevalence of mild cognitive impairment in type 2 diabetes mellitus patients: a systematic review and meta-analysis. Acta Diabetol. (2021) 58:671–85. doi: 10.1007/s00592-020-01648-9, PMID: 33417039

[3] Nimgampalle M Chakravarthy H Devanathan V . Chapter 8 - Glucose metabolism in the brain: An update. In: Viswanath B , editor. Recent Developments in Applied Microbiology and Biochemistry. San Diego, USA: Academic Press (2021). p. 77–88.

[4] Kim H-G . Cognitive dysfunctions in individuals with diabetes mellitus. Yeungnam Univ J Med. (2019) 36:183. doi: 10.12701/yujm.2019.00255, PMID: 31620632 PMC6784656

[5] Sharma G Parihar A Talaiya T Dubey K Porwal B Parihar MS . Cognitive impairments in type 2 diabetes, risk factors and preventive strategies. J Basic Clin Physiol Pharmacol. (2020) 31:20190105. doi: 10.1515/jbcpp-2019-0105, PMID: 31967962

[6] Xia SS Xia WL Huang JJ Zou HJ Tao J Yang Y . The factors contributing to cognitive dysfunction in type 2 diabetic patients. Ann Transl Med. (2020) 8:104. doi: 10.21037/atm.2019.12.113, PMID: 32175397 PMC7049020

[7] Rangaraju V Calloway N Ryan TA . Activity-driven local ATP synthesis is required for synaptic function. Cell. (2014) 156:825–35. doi: 10.1016/j.cell.2013.12.042, PMID: 24529383 PMC3955179

[8] Choi JH Kim MS . Homeostatic regulation of glucose metabolism by the central nervous system. Endocrinol Metab (Seoul). (2022) 37:9–25. doi: 10.3803/EnM.2021.1364, PMID: 35255598 PMC8901968

[9] Mergenthaler P Lindauer U Dienel GA Meisel A . Sugar for the brain: the role of glucose in physiological and pathological brain function. Trends Neurosci. (2013) 36:587–97. doi: 10.1016/j.tins.2013.07.001, PMID: 23968694 PMC3900881

[10] Steinbusch LKM Picard A Bonnet MS Basco D Labouèbe G Thorens B . Sex-specific control of fat mass and counterregulation by hypothalamic glucokinase. Diabetes. (2016) 65:2920–31. doi: 10.2337/db15-1514, PMID: 27422385

[11] Raju R Prabath I Chandrasekaran I Varadarajan S . Dorzagliatin: A breakthrough glucokinase activator coming on board to treat diabetes mellitus. Cureus. (2024) 16:e65708. doi: 10.7759/cureus.65708, PMID: 39211666 PMC11361462

[12] Li C Zhang Y Chen L Li X . Glucokinase and glucokinase activator. Life Metab. (2023) 2:load031. doi: 10.1093/lifemeta/load031, PMID: 39872624 PMC11749227

[13] Chen L Zhang J Sun Y Zhao Y Liu X Fang Z . A phase I open-label clinical trial to study drug-drug interactions of Dorzagliatin and Sitagliptin in patients with type 2 diabetes and obesity. Nat Commun. (2023) 14:1405. doi: 10.1038/s41467-023-36946-7, PMID: 36918550 PMC10014962

[14] Evans DM Davey Smith G . Mendelian randomization: new applications in the coming age of hypothesis-free causality. Annu Rev Genomics Hum Genet. (2015) 16:327–50. doi: 10.1146/annurev-genom-090314-050016, PMID: 25939054

[15] Akash MS Rehman K Chen S . Goto-Kakizaki rats: its suitability as non-obese diabetic animal model for spontaneous type 2 diabetes mellitus. Curr Diabetes Rev. (2013) 9:387–96. doi: 10.2174/15733998113099990069, PMID: 23855509

[16] Matsunaga Y Negishi T Hatakeyama A Kawagoe Y Sawano E Tashiro T . Impairment of synaptic development in the hippocampus of diabetic Goto-Kakizaki rats. Int J Dev Neurosci. (2016) 53:58–67. doi: 10.1016/j.ijdevneu.2016.07.004, PMID: 27444810

[17] Chen J Spracklen CN Marenne G Varshney A Corbin LJ Luan J . The trans-ancestral genomic architecture of glycemic traits. Nat Genet. (2021) 53:840–60. doi: 10.1038/s41588-021-00852-9, PMID: 34059833 PMC7610958

[18] Hatoum AS Morrison CL Mitchell EC Lam M Benca-Bachman CE Reineberg AE . Genome-wide association study shows that executive functioning is influenced by GABAergic processes and is a neurocognitive genetic correlate of psychiatric disorders. Biol Psychiatry. (2023) 93:59–70. doi: 10.1016/j.biopsych.2022.06.034, PMID: 36150907 PMC9722603

[19] Lee JJ Wedow R Okbay A Kong E Maghzian O Zacher M . Gene discovery and polygenic prediction from a genome-wide association study of educational attainment in 1.1 million individuals. Nat Genet. (2018) 50:1112–21. doi: 10.1038/s41588-018-0147-3, PMID: 30038396 PMC6393768

[20] Sakaue S Kanai M Tanigawa Y Karjalainen J Kurki M Koshiba S . A cross-population atlas of genetic associations for 220 human phenotypes. Nat Genet. (2021) 53:1415–24. doi: 10.1038/s41588-021-00931-x, PMID: 34594039 PMC12208603

[21] Pierce BL Ahsan H VanderWeele TJ . Power and instrument strength requirements for Mendelian randomization studies using multiple genetic variants. Int J Epidemiol. (2010) 40:740–52. doi: 10.1093/ije/dyq151, PMID: 20813862 PMC3147064

[22] Wald A . The fitting of straight lines if both variables are subject to error. Ann Math statistics. (1940) 11:284–300. doi: 10.1214/aoms/1177731868

[23] Burgess S Dudbridge F Thompson SG . Combining information on multiple instrumental variables in Mendelian randomization: comparison of allele score and summarized data methods. Stat Med. (2016) 35:1880–906. doi: 10.1002/sim.6835, PMID: 26661904 PMC4832315

[24] Bowden J Del Greco MF Minelli C Davey Smith G Sheehan NA Thompson JR . Assessing the suitability of summary data for two-sample Mendelian randomization analyses using MR-Egger regression: the role of the I2 statistic. Int J Epidemiol. (2016) 45:1961–74. doi: 10.1093/ije/dyw220, PMID: 27616674 PMC5446088

[25] Bowden J Davey Smith G Burgess S . Mendelian randomization with invalid instruments: effect estimation and bias detection through Egger regression. Int J Epidemiol. (2015) 44:512–25. doi: 10.1093/ije/dyv080, PMID: 26050253 PMC4469799

[26] Verbanck M Chen CY Neale B Do R . Detection of widespread horizontal pleiotropy in causal relationships inferred from Mendelian randomization between complex traits and diseases. Nat Genet. (2018) 50:693–8. doi: 10.1038/s41588-018-0099-7, PMID: 29686387 PMC6083837

[27] Nakrani MN Wineland RH Anjum F . Physiology, Glucose Metabolism. In: *StatPearls*. Treasure Island (FL): StatPearls Publishing; (2025). 32809434

[28] Han R Liang J Zhou B . Glucose metabolic dysfunction in neurodegenerative diseases—New mechanistic insights and the potential of hypoxia as a prospective therapy targeting metabolic reprogramming. Int J Mol Sci. (2021) 22:5887. doi: 10.3390/ijms22115887, PMID: 34072616 PMC8198281

[29] Bell SM Burgess T Lee J Blackburn DJ Allen SP Mortiboys H . Peripheral glycolysis in neurodegenerative diseases. Int J Mol Sci. (2020) 21:8924. doi: 10.3390/ijms21238924, PMID: 33255513 PMC7727792

[30] Dai C Tan C Zhao L Liang Y Liu G Liu H . Glucose metabolism impairment in Parkinson’s disease. Brain Res Bull. (2023) 199:110672. doi: 10.1016/j.brainresbull.2023.110672, PMID: 37210012

[31] Besson MT Alegría K Garrido-Gerter P Barros LF Liévens JC . Enhanced neuronal glucose transporter expression reveals metabolic choice in a HD Drosophila model. PLoS One. (2015) 10:e0118765. doi: 10.1371/journal.pone.0118765, PMID: 25761110 PMC4356621

[32] Stranahan AM . Models and mechanisms for hippocampal dysfunction in obesity and diabetes. Neuroscience. (2015) 309:125–39. doi: 10.1016/j.neuroscience.2015.04.045, PMID: 25934036 PMC4624614

[33] Xu W Wang X Hou X Yang Y Ma R Lv R . The role of microglia in the pathogenesis of diabetic-associated cognitive dysfunction. Front Endocrinol. (2024) 14:1246979. doi: 10.3389/fendo.2023.1246979, PMID: 38274227 PMC10808430

[34] He S Liang Q Zhu J Wang C Lin X Wu D . Research landscape and emerging trends of diabetes-associated cognitive dysfunction: a bibliometric analysis. Front Neurosci. (2023) 17:1214301. doi: 10.3389/fnins.2023.1214301, PMID: 37575299 PMC10416239

[35] Matschinsky FM Wilson DF . The central role of glucokinase in glucose homeostasis: A perspective 50 years after demonstrating the presence of the enzyme in islets of Langerhans. Front Physiol. (2019) 10:148. doi: 10.3389/fphys.2019.00148, PMID: 30949058 PMC6435959

[36] Zhu D Gan S Liu Y Ma J Dong X Song W . Dorzagliatin monotherapy in Chinese patients with type 2 diabetes: a dose-ranging, randomised, double-blind, placebo-controlled, phase 2 study. Lancet Diabetes Endocrinol. (2018) 6:627–36. doi: 10.1016/S2213-8587(18)30105-0, PMID: 29735394

[37] Chow E Wang K Lim CK Tsoi ST Fan B Poon E . Dorzagliatin, a dual-acting glucokinase activator, increases insulin secretion and glucose sensitivity in glucokinase maturity-onset diabetes of the young and recent-onset type 2 diabetes. Diabetes. (2023) 72:299–308. doi: 10.2337/db22-0708, PMID: 36342518 PMC9871194

[38] Li W Su L Li M Xu J . Efficacy of Dorzagliatin combined with metformin in the treatment of type 2 diabetes mellitus complicated with mild cognitive impairment. J Xinjiang Med University. (2024) 47:1458–63.

[39] Wang P Liu H Chen L Duan Y Chen Q Xi S . Effects of a novel glucokinase activator, HMS5552, on glucose metabolism in a rat model of type 2 diabetes mellitus. J Diabetes Res. (2017) 2017:5812607. doi: 10.1155/2017/5812607, PMID: 28191470 PMC5278194

[40] Yoon JH Hwang J Son SU Choi J You S-W Park H . How can insulin resistance cause Alzheimer’s disease? Int J Mol Sci. (2023) 24:3506. doi: 10.3390/ijms24043506, PMID: 36834911 PMC9966425

[41] Arrieta-Cruz I Gutiérrez-Juárez R . The role of insulin resistance and glucose metabolism dysregulation in the development of Alzheimer´s disease. Rev Invest Clin. (2016) 68:53–8. doi: 10.1016/S0034-8376(25)00208-6, PMID: 27103040

[42] García-Cáceres C Quarta C Varela L Gao Y Gruber T Legutko B . Astrocytic insulin signaling couples brain glucose uptake with nutrient availability. Cell. (2016) 166:867–80. doi: 10.1016/j.cell.2016.07.028, PMID: 27518562 PMC8961449

[43] Soto M Cai W Konishi M Kahn CR . Insulin signaling in the hippocampus and amygdala regulates metabolism and neurobehavior. Proc Natl Acad Sci U.S.A. (2019) 116:6379–84. doi: 10.1073/pnas.1817391116, PMID: 30765523 PMC6442573

[44] Gali CC Fanaee-Danesh E Zandl-Lang M Albrecher NM Tam-Amersdorfer C Stracke A . Amyloid-beta impairs insulin signaling by accelerating autophagy-lysosomal degradation of LRP-1 and IR-β in blood-brain barrier endothelial cells *in vitro* and in 3XTg-AD mice. Mol Cell Neurosci. (2019) 99:103390. doi: 10.1016/j.mcn.2019.103390, PMID: 31276749 PMC6897558

[45] Sędzikowska A Szablewski L . Insulin and insulin resistance in Alzheimer’s disease. Int J Mol Sci. (2021) 22:9987. doi: 10.3390/ijms22189987, PMID: 34576151 PMC8472298

[46] Szablewski L . Brain glucose transporters: role in pathogenesis and potential targets for the treatment of Alzheimer’s disease. Int J Mol Sci. (2021) 22:8142. doi: 10.3390/ijms22158142, PMID: 34360906 PMC8348194

[47] Albaik M Sheikh Saleh D Kauther D Mohammed H Alfarra S Alghamdi A . Bridging the gap: glucose transporters, Alzheimer’s, and future therapeutic prospects. Front Cell Dev Biol. (2024) 12:1344039. doi: 10.3389/fcell.2024.1344039, PMID: 38298219 PMC10824951

[48] Simpson IA Vannucci SJ Maher F . Glucose transporters in mammalian brain. Biochem Soc Trans. (1994) 22:671–5. doi: 10.1042/bst0220671, PMID: 7821661

[49] Harr SD Simonian NA Hyman BT . Functional alterations in Alzheimer’s disease: decreased glucose transporter 3 immunoreactivity in the perforant pathway terminal zone. J Neuropathol Exp Neurol. (1995) 54:38–41. doi: 10.1097/00005072-199501000-00005, PMID: 7815078

[50] Liu Y Liu F Iqbal K Grundke-Iqbal I Gong C-X . Decreased glucose transporters correlate to abnormal hyperphosphorylation of tau in Alzheimer disease. FEBS letters. (2008) 582:359–64. doi: 10.1016/j.febslet.2007.12.035, PMID: 18174027 PMC2247364

[51] Hanson JE Yuan H Perszyk RE Banke TG Xing H Tsai M-C . Therapeutic potential of N-methyl-D-aspartate receptor modulators in psychiatry. Neuropsychopharmacology. (2024) 49:51–66. doi: 10.1038/s41386-023-01614-3, PMID: 37369776 PMC10700609

[52] Newcomer JW Farber NB Olney JW . NMDA receptor function, memory, and brain aging. Dialogues Clin Neurosci. (2000) 2:219–32. doi: 10.31887/DCNS.2000.2.3/jnewcomer, PMID: 22034391 PMC3181613

[53] Dickson BJ . Wiring the brain with insulin. Science. (2003) 300:440–1. doi: 10.1126/science.1084513, PMID: 12702863

[54] Kremerskothen J Wendholt D Teber I Barnekow A . Insulin-induced expression of the activity-regulated cytoskeleton-associated gene (ARC) in human neuroblastoma cells requires p21ras, mitogen-activated protein kinase/extracellular regulated kinase and src tyrosine kinases but is protein kinase C-independent. Neurosci Letters. (2002) 321:153–6. doi: 10.1016/S0304-3940(01)02532-0, PMID: 11880195

[55] Ference BA . How to use Mendelian randomization to anticipate the results of randomized trials. Eur Heart J. (2018) 39:360–2. doi: 10.1093/eurheartj/ehx462, PMID: 29020392

