# Supplementary material for: Dorzagliatin shows potential in preventing cognitive impairment in diabetes: evidence from Mendelian randomization analysis and animal study
Source: Front Endocrinol (Lausanne). 2026 Jan 23;16:1755359. doi: 10.3389/fendo.2025.1755359 (PMC12875913; doi:10.3389/fendo.2025.1755359)
Supplement: Supplementary file 1 [file DataSheet1.docx]

**Supplementary materials**


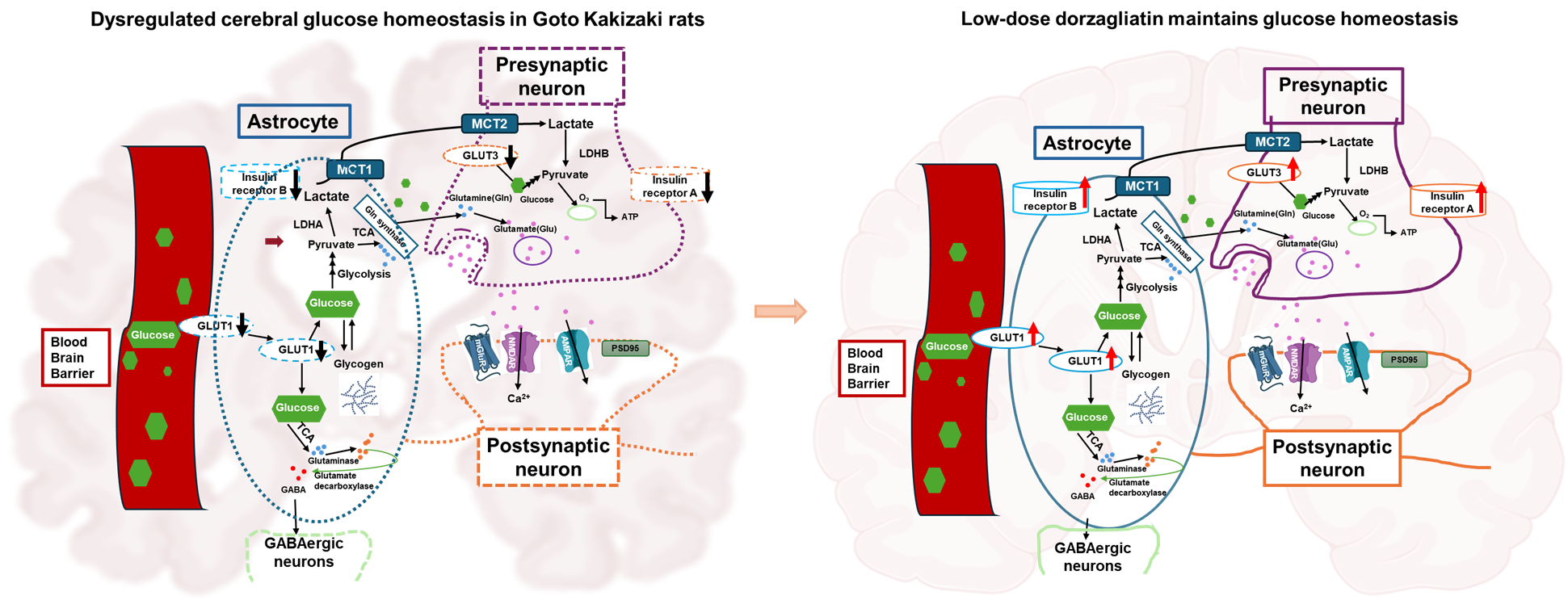


**Figure S1. The role of dorzagliatin in neuroprotective effects in diabetic rats.** In Goto–Kakizaki rats, a chronic imbalance in glucose homeostasis leads to downregulated GLUT1/3 expression, reduced neuronal glucose uptake, and impaired glycolytic flux. This flux is critical for both ATP generation and providing carbon backbones for *de novo* glutamate/GABA synthesis. This dual energetic–precursor deficit destabilised NMDA receptor-dependent synaptic plasticity and results in spatial memory impairment. Low-dose dorzagliatin treatment protects against memory loss by improving glycemic control, potentially through maintenance of cerebral glucose homeostasis and neurotransmitter synthesis via preservation of glucose transporters (GLUT1 and GLUT3) expression, enhancement of insulin signaling pathways, and support of NMDA receptor-mediated synaptic transmission.

Table S1. Information on the investigated traits.

| Trait | Participants | Web source and publication | Brief trait definition |
| --- | --- | --- | --- |
| Type 2 diabetes | 38,841 European cases and 451,248 European controls | <https://gwas.mrcieu.ac.uk/datasets/ebi-a-GCST90018926/>  PMID: 34594039 | ICD-10 code: E11 |
| Memory loss | 1,384 European cases and 210,333 European controls | <https://gwas.mrcieu.ac.uk/datasets/finn-b-MEMLOSS/>  <https://r5.finngen.fi/pheno/MEMLOSS> | ICD-10 code: R41.1 (Anterograde amnesia)  ICD-10 code: R41.2 (Retrograde amnesia)  ICD-10 code: R41.3 (Other amnesia) |
| Prospective memory task | 162,335 Europeans | <https://www.ebi.ac.uk/gwas/studies/GCST90179116>  PMID: 36150907 | Factor score based on the “touch shape test”. See UKB data field 20018 for details of the test. |
| Symbol digit substitution task | 84,125 Europeans | <https://www.ebi.ac.uk/gwas/studies/GCST90179119>  PMID: 36150907 | Factor score based on the “symbol-digit matching test”. See UKB data field 20159 for details of the test. |
| Intelligence | 216,381 Europeans | <https://www.ebi.ac.uk/gwas/studies/GCST90179114>  PMID: 36150907 | Factor score based on the “fluid intelligence/ reasoning” test. See UKB data field 20016 and 20191 for details of the test. |
| Cognitive performance | 257,841 Europeans | <https://gwas.mrcieu.ac.uk/datasets/ebi-a-GCST006572/>  PMID: 30038396 | UKB: Standardized score on a test of verbal-numerical reasoning (data field 20016 for in-person assessments and data field 20191 for an online follow-up).  COGENT cohort: Standardized score on the first unrotated component of the performance of at least 3 neuropsychological tests (or at least two IQ-test subscales). |

Table S2. Instrumental variables for genetically mimicked glucokinase activation in the MAGIC study.

| SNP | Position | Effect allele | Other allele | EAF | Effect size | P |
| --- | --- | --- | --- | --- | --- | --- |
| rs2595701 | 7:44148553 | A | G | 0.317 | 0.010 | 4.8E-11 |
| rs61736256 | 7:44153614 | C | G | 0.964 | -0.023 | 3.0E-09 |
| rs2908277 | 7:44183433 | A | G | 0.117 | 0.017 | 1.3E-18 |
| rs28684786 | 7:44206824 | T | C | 0.901 | 0.020 | 8.1E-10 |
| rs1303722 | 7:44219074 | T | C | 0.463 | -0.012 | 1.9E-23 |
| rs2300584 | 7:44219338 | A | G | 0.751 | -0.023 | 1.2E-55 |
| rs2284769 | 7:44222220 | C | G | 0.889 | 0.013 | 7.5E-09 |
| rs2971670 | 7:44226101 | T | C | 0.181 | 0.032 | 5.1E-88 |
| rs74897641 | 7:44230381 | A | G | 0.013 | 0.047 | 3.0E-11 |
| rs3757840 | 7:44231216 | T | G | 0.52 | 0.022 | 4.3E-71 |
| rs138917529 | 7:44235694 | A | T | 0.983 | 0.038 | 9.5E-11 |
| rs79784692 | 7:44256526 | A | G | 0.933 | 0.025 | 8.2E-15 |
| rs2075067 | 7:44263028 | A | C | 0.15 | 0.011 | 1.5E-08 |
| rs10951758 | 7:44266077 | A | G | 0.526 | -0.013 | 6.5E-20 |
| rs59374739 | 7:44266570 | T | G | 0.727 | -0.018 | 6.8E-22 |
| rs4724295 | 7:44300964 | A | G | 0.627 | 0.009 | 3.1E-09 |
| rs66482342 | 7:44319048 | T | C | 0.086 | 0.018 | 1.4E-09 |

| Exposure | Outcome | Test | P value |
| --- | --- | --- | --- |
| Genetically mimicked glucokinase activation instrumented by 17 *GCK* SNPs (per 1 % lower HbA1c) | Memory loss | Heterogeneity | P_Q-statistic_ =0.915 |
|  |  | Horizontal pleiotropy | P_egger-intercept_ =0.841 |
|  |  | Outlier | P_global-test_ =0.926 |
|  | Prospective memory task | Heterogeneity | P_Q-statistic_ =0.634 |
|  |  | Horizontal pleiotropy | P_egger-intercept_ =0.329 |
|  |  | Outlier | P_global-test_ =0.609 |
|  | Symbol digit substitution task | Heterogeneity | P_Q-statistic_ =0.759 |
|  |  | Horizontal pleiotropy | P_egger-intercept_ =0.808 |
|  |  | Outlier | P_global-test_ =0.759 |
|  | Intelligence | Heterogeneity | P_Q-statistic_ =0.710 |
|  |  | Horizontal pleiotropy | P_egger-intercept_ =0.848 |
|  |  | Outlier | P_global-test_ =0.737 |

Table S3. Sensitivity tests for significant associations in the MR analyses

Table S4. Instrumental variables for genetically predicted lower HbA1c in the MAGIC study.

| SNP | Position | Effect allele | Other allele | EAF | Effect size | Standard error | P |
| --- | --- | --- | --- | --- | --- | --- | --- |
| rs267738 | 1:150940625 | T | G | 0.797 | 0.0109 | 0.0016 | 1.14E-11 |
| rs857725 | 1:158607935 | T | G | 0.723 | -0.0208 | 0.0014 | 5.43E-55 |
| rs7547793 | 1:203653544 | A | C | 0.12 | -0.0118 | 0.0021 | 6.61E-09 |
| rs340882 | 1:214145731 | C | G | 0.42 | -0.0084 | 0.0013 | 1.48E-10 |
| rs2375278 | 1:25529038 | A | G | 0.176 | 0.0112 | 0.0017 | 1.05E-11 |
| rs1175549 | 1:3691727 | A | C | 0.786 | 0.0098 | 0.0015 | 7.13E-13 |
| rs560887 | 2:169763148 | T | C | 0.306 | -0.0307 | 0.0014 | 5.55E-122 |
| rs13389076 | 2:169789512 | A | G | 0.034 | 0.0332 | 0.0038 | 3.04E-18 |
| rs13419763 | 2:219134950 | T | C | 0.588 | 0.008 | 0.0014 | 5.48E-09 |
| rs12612492 | 2:24093756 | T | C | 0.148 | 0.0188 | 0.0019 | 1.88E-26 |
| rs1367173 | 2:43449385 | T | C | 0.106 | -0.0152 | 0.002 | 1.66E-14 |
| rs79403657 | 2:48114094 | C | G | 0.823 | -0.009 | 0.0017 | 2.03E-08 |
| rs10169706 | 2:5791194 | T | C | 0.04 | 0.026 | 0.0046 | 1.48E-08 |
| rs12491937 | 3:12268244 | A | G | 0.555 | 0.009 | 0.0013 | 1.42E-13 |
| rs11719201 | 3:123068744 | T | C | 0.182 | -0.0129 | 0.0015 | 2.43E-18 |
| rs6804915 | 3:170627909 | A | C | 0.288 | -0.0108 | 0.0014 | 2.76E-16 |
| rs13089972 | 3:171798694 | A | T | 0.584 | 0.0111 | 0.0014 | 1.87E-15 |
| rs9818758 | 3:49382925 | A | G | 0.204 | 0.0131 | 0.0017 | 1.49E-13 |
| rs6798941 | 3:52893465 | T | C | 0.322 | 0.0086 | 0.0015 | 1.49E-08 |
| rs13134327 | 4:144659795 | A | G | 0.331 | 0.0144 | 0.0014 | 2.81E-26 |
| rs6877043 | 5:154048367 | T | C | 0.638 | 0.0085 | 0.0014 | 1.99E-10 |
| rs9376090 | 6:135411228 | T | C | 0.728 | 0.0247 | 0.0014 | 1.90E-62 |
| rs10946402 | 6:20715826 | T | G | 0.831 | -0.0101 | 0.0016 | 1.12E-10 |
| rs1800562 | 6:26093141 | A | G | 0.046 | -0.0383 | 0.0027 | 2.33E-50 |
| rs204995 | 6:32154285 | A | G | 0.781 | -0.0098 | 0.0018 | 1.93E-09 |
| rs3778321 | 6:7250270 | A | G | 0.176 | -0.0106 | 0.0016 | 4.18E-11 |
| rs4727979 | 7:123429697 | A | C | 0.906 | 0.0121 | 0.0024 | 4.61E-08 |
| rs10231021 | 7:15060429 | A | T | 0.492 | 0.0089 | 0.0013 | 8.69E-14 |
| rs13234131 | 7:73025975 | A | G | 0.876 | -0.0113 | 0.002 | 2.06E-09 |
| rs11558471 | 8:118185733 | A | G | 0.707 | 0.0151 | 0.0014 | 3.38E-25 |
| rs2001846 | 8:126478450 | T | C | 0.471 | -0.0069 | 0.0013 | 8.58E-10 |
| rs6474359 | 8:41549194 | T | C | 0.978 | 0.0427 | 0.0038 | 1.91E-33 |
| rs4737009 | 8:41630405 | A | G | 0.262 | 0.0228 | 0.0015 | 8.29E-56 |
| rs7042939 | 9:110511408 | A | G | 0.418 | 0.0102 | 0.0013 | 1.50E-15 |
| rs651007 | 9:136153875 | T | C | 0.215 | 0.0108 | 0.0015 | 3.28E-15 |
| rs3829109 | 9:139256766 | A | G | 0.276 | -0.0086 | 0.0015 | 2.68E-08 |
| rs10811661 | 9:22134094 | T | C | 0.835 | 0.0128 | 0.0017 | 1.74E-14 |
| rs7861647 | 9:79977386 | T | C | 0.193 | 0.0128 | 0.0016 | 4.50E-14 |
| rs61750929 | 9:91495135 | T | C | 0.041 | -0.0284 | 0.0029 | 9.49E-24 |
| rs7903146 | 10:114758349 | T | C | 0.307 | 0.0133 | 0.0014 | 1.04E-22 |
| rs11257655 | 10:12307894 | T | C | 0.241 | 0.011 | 0.0016 | 1.91E-13 |
| rs2102339 | 10:71015389 | T | C | 0.334 | -0.0087 | 0.0014 | 3.42E-10 |
| rs16926246 | 10:71093392 | T | C | 0.136 | -0.0727 | 0.0021 | 1.00E-200 |
| rs7127313 | 11:100508897 | T | C | 0.336 | 0.0066 | 0.0013 | 4.85E-08 |
| rs608793 | 11:118986659 | T | C | 0.479 | 0.0065 | 0.0013 | 4.55E-08 |
| rs4980325 | 11:234451 | T | G | 0.532 | 0.0108 | 0.0014 | 4.70E-14 |
| rs11039154 | 11:47278502 | T | C | 0.277 | -0.0087 | 0.0014 | 3.11E-09 |
| rs174559 | 11:61581656 | A | G | 0.285 | -0.0106 | 0.0014 | 3.31E-13 |
| rs10830963 | 11:92708710 | C | G | 0.714 | -0.0197 | 0.0015 | 1.54E-36 |
| rs360147 | 11:9790817 | T | C | 0.264 | -0.0086 | 0.0015 | 2.08E-09 |
| rs10774624 | 12:111833788 | A | G | 0.525 | 0.0093 | 0.0013 | 4.17E-14 |
| rs117233107 | 12:4328521 | A | G | 0.02 | -0.047 | 0.0072 | 8.45E-11 |
| rs4760682 | 12:48512285 | A | C | 0.817 | 0.0164 | 0.0018 | 3.20E-20 |
| rs76533333 | 13:113352916 | A | G | 0.913 | -0.0265 | 0.0025 | 2.81E-29 |
| rs1278769 | 13:113536627 | A | G | 0.231 | -0.0091 | 0.0015 | 5.52E-12 |
| rs1535464 | 14:100793431 | A | G | 0.212 | -0.0086 | 0.0017 | 1.11E-08 |
| rs151165 | 14:65272626 | A | T | 0.397 | 0.0079 | 0.0014 | 2.04E-09 |
| rs10151436 | 14:73616095 | A | T | 0.89 | 0.013 | 0.0021 | 3.85E-11 |
| rs452306 | 15:65822777 | T | C | 0.627 | -0.0098 | 0.0014 | 5.51E-13 |
| rs11643024 | 16:11443183 | A | G | 0.303 | 0.0084 | 0.0015 | 7.98E-10 |
| rs7190771 | 16:28590030 | A | G | 0.332 | 0.0085 | 0.0013 | 6.02E-11 |
| rs11248914 | 16:293562 | T | C | 0.698 | 0.0114 | 0.0014 | 1.42E-14 |
| rs7198799 | 16:68818390 | T | C | 0.281 | 0.0083 | 0.0014 | 4.76E-09 |
| rs837763 | 16:88853729 | T | C | 0.578 | 0.0176 | 0.0013 | 5.20E-38 |
| rs9914988 | 17:27183104 | A | G | 0.802 | 0.0125 | 0.0016 | 4.66E-17 |
| rs2748427 | 17:76121864 | A | G | 0.803 | -0.0307 | 0.0022 | 9.82E-49 |
| rs9909940 | 17:80689036 | T | C | 0.323 | 0.0322 | 0.0014 | 1.43E-116 |
| rs28671200 | 18:43774444 | T | G | 0.646 | 0.0086 | 0.0017 | 1.56E-08 |
| rs17533945 | 19:17257802 | T | C | 0.582 | -0.0128 | 0.0014 | 1.62E-23 |
| rs10405535 | 19:33072085 | A | G | 0.29 | 0.0122 | 0.0016 | 6.47E-14 |
| rs737092 | 20:55990405 | T | C | 0.501 | -0.0073 | 0.0013 | 7.57E-09 |
| rs855791 | 22:37462936 | A | G | 0.4 | 0.0188 | 0.0013 | 1.34E-56 |
| rs8138197 | 22:43114551 | A | G | 0.488 | -0.0073 | 0.0014 | 3.54E-08 |
